# Corrigendum to “Do Hearing Aids Address Real-World Hearing Difficulties for Adults With Mild Hearing Impairment? Results From a Pilot Study Using Ecological Momentary Assessment.”

**DOI:** 10.1177/23312165221121305

**Published:** 2022-08-22

Timmer, B.H.B., Hickson, L., & Launer, S. (January 2018). Do Hearing Aids Address Real-World Hearing Difficulties for Adults With Mild Hearing Impairment? Results From a Pilot Study Using Ecological Momentary Assessment. *Trends in Hearing*. doi:10.1177/2331216518783608

In the above article Michael David is to be included as third author with the affiliation as Louise Hickson – School of Health and Rehabilitation Sciences, The University of Queensland, Australia.

Hence, the author list will be,

Barbra H. B. Timmer, Louise Hickson, Michael David, Stefan Launer

